# Identification of Important N-Linked Glycosylation Sites in the Hemagglutinin Protein and Their Functional Impact on DC-SIGN Mediated Avian Influenza H5N1 Infection

**DOI:** 10.3390/ijms22020743

**Published:** 2021-01-13

**Authors:** Zih-Syuan Yang, Szu-Wei Huang, Wen-Hung Wang, Chih-Yen Lin, Chu-Feng Wang, Aspiro Nayim Urbina, Arunee Thitithanyanont, Sung-Pin Tseng, Po-Liang Lu, Yen-Hsu Chen, Sheng-Fan Wang

**Affiliations:** 1 Center for Tropical Medicine and Infectious Disease, Kaohsiung Medical University, Kaohsiung 80708, Taiwan; r99100125@gmail.com (Z.-S.Y.); bole0918@gmail.com (W.-H.W.); pigpipi831205@gmail.com (C.-Y.L.); aspiro.urbina@hotmail.com (A.N.U.); idpaul@gmail.com (P.-L.L.); d810070@kmu.edu.tw (Y.-H.C.); 2 Department of Medical Laboratory Science and Biotechnology, Kaohsiung Medical University, Kaohsiung 80708, Taiwan; tsengsp@kmu.edu.tw; 3 Model Development Section, Basic Research Laboratory, Center for Cancer Research, National Cancer Institute, Frederick, MD 21702, USA; szu-wei.huang@nih.gov; 4 Division of Infectious Disease, Department of Internal Medicine, Kaohsiung Medical, University Hospital, Kaohsiung Medical University, Kaohsiung 80708, Taiwan; 5 Clinical Microbiology Laboratory, Department of Laboratory Medicine, Kaohsiung Medical University Hospital, Kaohsiung Medical University, Kaohsiung 80708, Taiwan; virus7047@gmail.com; 6 Department of Microbiology, Faculty of Science, Mahidol University, Bangkok 10400, Thailand; arunee.thi@mahidol.ac.th; 7 Department of Medical Research, Kaohsiung Medical University Hospital, Kaohsiung Medical University, Kaohsiung 80708, Taiwan

**Keywords:** DC-SIGN, H5N1, N27Q, N39Q, hemagglutinin, N-linked glycosylation, infection

## Abstract

DC-SIGN, a C-type lectin mainly expressed in dendritic cells (DCs), has been reported to mediate several viral infections. We previously reported that DC-SIGN mediated H5N1 influenza A virus (AIVs) infection, however, the important DC-SIGN interaction with N-glycosylation sites remain unknown. This study aims to identify the optimal DC-SIGN interacting N-glycosylation sites in HA proteins of H5N1-AIVs. Results from NetNGlyc program analyzed the H5 hemagglutinin sequences of isolates during 2004–2020, revealing that seven and two conserved N-glycosylation sites were detected in HA1 and HA2 domain, respectively. A lentivirus pseudotyped A/Vietnam/1203/04 H5N1 envelope (H5N1-PVs) was generated which displayed an abundance of HA5 proteins on the virions via immuno-electron microscope observation. Further, H5N1-PVs or reverse-genetics (H5N1-RG) strains carrying a serial N-glycosylated mutation was generated by site-directed mutagenesis assay. Human recombinant DC-SIGN (rDC-SIGN) coated ELISA showed that H5N1-PVs bound to DC-SIGN, however, mutation on the N27Q, N39Q, and N181Q significantly reduced this binding (*p* < 0.05). Infectivity and capture assay demonstrated that N27Q and N39Q mutations significantly ameliorated DC-SIGN mediated H5N1 infection. Furthermore, combined mutations (N27Q&N39Q) significantly waned the interaction on either H5N1-PVs or -RG infection in *cis* and in *trans* (*p* < 0.01). This study concludes that N27 and N39 are two essential N-glycosylation contributing to DC-SIGN mediating H5N1 infection.

## 1. Introduction

The first reported human infected with the highly pathogenic avian influenza (HPAI) H5N1 virus was recorded in Hong Kong, 1997 [1]. Subsequently, the spread of HPAI H5N1 viruses in poultry and sporadic human infections are perceived as a potential pandemic threat [2,3]. At present, there are several clades and subclades being identified based on their genetic and antigenic variation of the viral haemagglutinin (HA5) and neuraminidase (NA1) genes. Phylogenetic analysis indicates that some of the 10 first-order clades (0–9) have ceased to circulate since 2008 or even earlier (clades 0, 3, 4, 5, 6, 8, 9), as have some second- and third-order groups of clades 2. Meanwhile, clades 1, 2.1.3, 2.2, 2.2.1, 2.3.2, 2.3.4, and 7 have continued to evolve [4]. Among them, clade 2.3.2 is widely distributed in Asian countries. Most H5N1 human infection results from direct contact with infected poultry [5]. Between 2003 and October 2020, there have been 861 human cases and 455 deaths reported by the World Health Organization (WHO) [6].

Influenza A viruses belong to the Orthomyxoviridae family. Their genome consists of eight negative stranded RNA segments, which encode for more than 10 major proteins [7]. The influenza A virion is studded with glycoprotein spikes of hemagglutinin (HA) and neuraminidase (NA), in a ratio of approximately four to one, projecting from a host cell-derived lipid membrane [8]. In addition, a smaller number of matrix (M2) ion channels traverse the lipid envelope. The envelope and its three integral membrane proteins HA, NA, and M2 overlay a matrix of M1 protein, which encloses the virion core [9]. The attachment of influenza A virus to sialic acids (SAs) on the cell surface is a critical first step in the initiation of infection. SAs are an essential factor for the tropism of the influenza virus since their type of linkage to the galactose residue determines whether they are recognized by the specific viruses. SAs are widely expressed in most tissues and organs. Avian influenza A viruses (AIVs) have a preference in recognizing sialo-sugar chains terminating in sialic acid-α2,3-galactose (SAα2,3Gal), whereas human influenza A viruses prefer SAα2,6Gal. Regarding the pathogenesis of H5N1, regardless of the exposure route, the majority of cases develop viral pneumonia [3]. During the initial phase of human H5N1 infection, the viruses are rarely isolated from the upper respiratory tract, most likely due to high abundance of SAα2,3Gal on the cells of the lower respiratory tract in humans as opposed to the upper respiratory tract [10]. The cases of H5N1 infection have recorded a high mortality rate (~60%) [3,11]. The disease severity of H5N1 in human may be induced by several factors, such as virulence, strain type, cytokine storm, and immune dysregulation [12]. In addition to lung dysfunction, systemic infection and multiple organ failure are often observed in the H5N1 fatal cases [13,14,15]. However, the detailed mechanism regarding severe H5N1 infection remains unclear.

In recent decades, lectins have proven to play critical roles in regulation of microorganisms’ infection [16,17,18,19,20]. DC-SIGN (dendritic cell-specific ICAM-3-grabbing nonintegrin) is a calcium-dependent lectin, with a wide range of biological functions. High-level expression of DC-SIGN has been demonstrated on immature DC and macrophage subpopulations abundant in the dermis of the skin, at mucosal surfaces, and in lymph nodes and peripheral tissues [21]. In addition to regulate DC migration and T-lymphocyte activation via interaction with ICAM-3, expression of DC-SIGN has been reported to mediate and enhance many viral infections, such as HIV-1 [22], HCV [23], Ebola virus [24], dengue virus [25], Coronavirus [26], and influenza virus [27,28,29]. DC-SIGN has been reported to interact with specific carbohydrate structures on pathogens to internalize pathogens via clathrin-mediated endocytosis for degradation in lysosomal compartments to enhance antigen processing and presentation [21]. Regarding HIV-1, DC-SIGN binds to the highly glycosylated HIV envelope (Env) gp120 in a CD4-independent fashion and can efficiently transfer the virus to CD4+ permissive T cells, thereby facilitating viral infection in *tran*s [22,30,31].

Influenza HA is a highly glycosylated protein [32,33,34]. Cellular lectin receptors may recognize the glycans on HA of influenza A virus, allowing for the binding of the virus to the cells and permitting internalization. Thus, the extent of glycosylation of HA is likely important for the recognition of the virus by cellular lectins. Glycosylation is achieved by post translational modification of Asparagine residues of the NXS/T motif (X can be any amino acid except Proline) [33,34]. Numbers, types, and the positions of glycans vary for each virus, which might affect recognition of influenza viruses by the lectin receptors such as DC-SIGN. We were the first to reported that DC-SIGN acts as an attachment molecule which facilitates H5N1 infection, in addition to assist in H5N1 dissemination to the other susceptible cells [29]. Following our findings, several studies indicate that DC-SIGN plays an important role in H5N1 infection and H5N1 pathogenesis [27,35,36]. Furthermore, Londrigan et al. even reported DC-SIGN as an alternative receptor for influenza H1N1 entry [28] and Hillaire et al. reported that in the absence of sialic acids, human influenza A viruses can replicate in DC-SIGN expressing cells, indicating that efficiency of DC-SIGN mediated infection is dependent on the extent of glycosylation of the viral hemagglutinin [27].

Currently, the detail mechanism of severe pathogenesis caused by H5N1 AIVs infection remains unclear. We proposed that the interaction between DC-SIGN and HA may play an important role in regulation of H5N1 AIVs infection and transmission. The essential N-linked glycosylation sites on HA are the main factors to modulate this interaction. In this study, we aimed to identify important DC-SIGN interaction with the N-linked glycosylation sites on HA of H5N1 AIVs.

## 2. Results

### 2.1. N-Linked Glycosylation Sites Are Conserved in HA of H5N1 AIVs among Different Clades Phylogenetically

**Figure 1:**
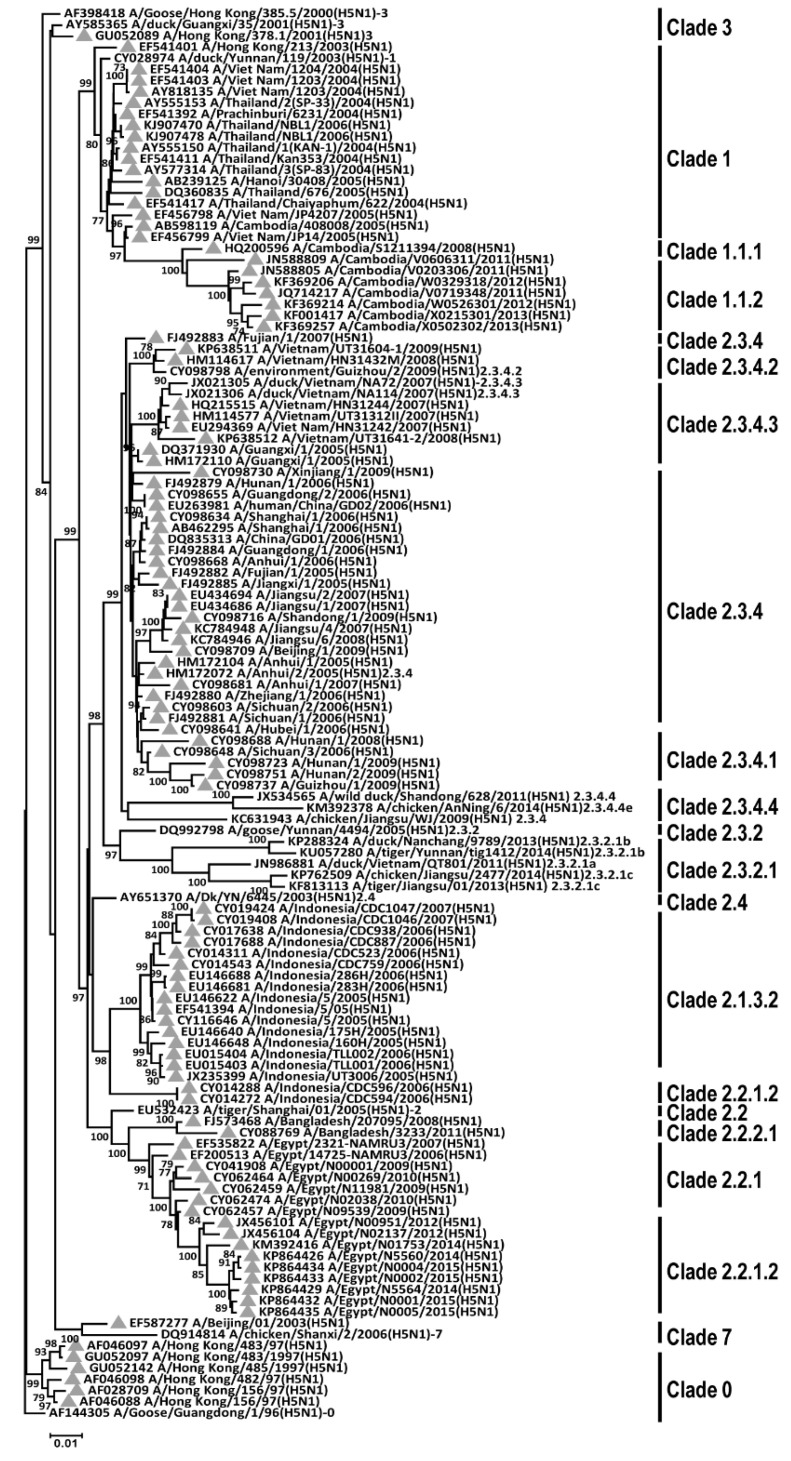
Phylogenetic analysis of the HA gene of avian H5N1 influenza viruses. The full-length of HA nucleotide sequences from different clades of avian H5N1 viruses were obtained via NCBI Influenza Virus Resource. The nucleotide sequences of complete HA-genes of avian H5N1 viruses were aligned, edited, and analyzed using ClustalW software. The phylogenetic analysis was performed using MEGA version X (http://www.megasoftware.net/). Consensus neighbor-joining trees were obtained from 1000 bootstrap replicates. The grey-filled triangles indicate avian H5N1 strains isolated from human.

**Figure 2:**
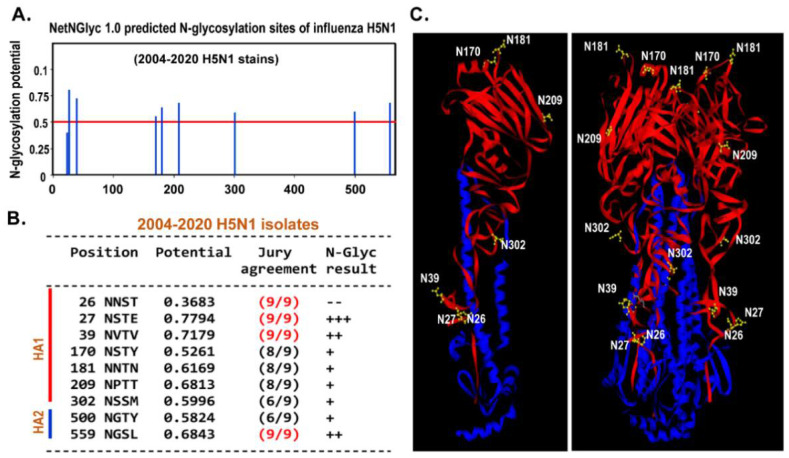
N-linked glycosylation analysis of HA protein of influenza H5N1 viruses. The full-length of HA nucleotide sequences form avian influenza H5N1 viruses isolated from infected persons during 2004–2020. The sequences were subjected to prediction of N-linked glycosylation on hemagglutinin of H5N1 influenza strain via by NetNGlyc 1.0 Server. (**A**) A total of nine N-linked glycosylation sites were detected. The red and blue lines indicate the potential threshold of N-linked glycosylation and predicted N-glycosylation sites on HA(H5), respectively. (**B**) There were seven N-glycosylation sites (26, 27, 39, 170, 181, 209, 302) located in HA1 domain, where two (500 and 509) were located in HA2 transmembrane domain. The red words indicate the highest percentage and scores of predicted N-linked glycosylation. (**C**) Each N-glycosylation site on the hemagglutinin structure was labeled; numbers indicate relative amino acid site. The left and right indicate monomeric and trimeric structure of the hemagglutinin of H5N1 influenza viruses. The red and blue structures indicate HA1 and HA2 domain, respectively.

Firstly, the N-linked glycosylation pattern on HA of H5N1 AIVs were analyzed. The different clades of avian H5N1 isolated from human under phylogenetic analysis were selected (Figure 1). These human H5N1 isolates were subjected to N-glycosylation prediction. The results predicted that there was a total of nine N-glycosylations on HA. Among these N-glycosylations, there were seven (amino acid residues N26, N27, N39, N170, N181, N209, and N302) and two (residues N500 and N559) N-linked glycosylation sites predicted to be express on HA1 and HA2 domain, respectively (Figure 2A,B). The N26, N27, N39, N302 were located on the side of HA, whereas the N170, N181, and N209 were distributed on the top of HA (Figure 2C). It was noted that during 2004–2020, these N-glycosylation sites were conserved suggesting that these conserved N-glycosylations might play certain essential roles in H5N1 AIVs’ life cycle (Figure 2).

### 2.2. Generation of Recombinant DC-SIGN Proteins for Interaction with H5N1 Virus

**Figure 3:**
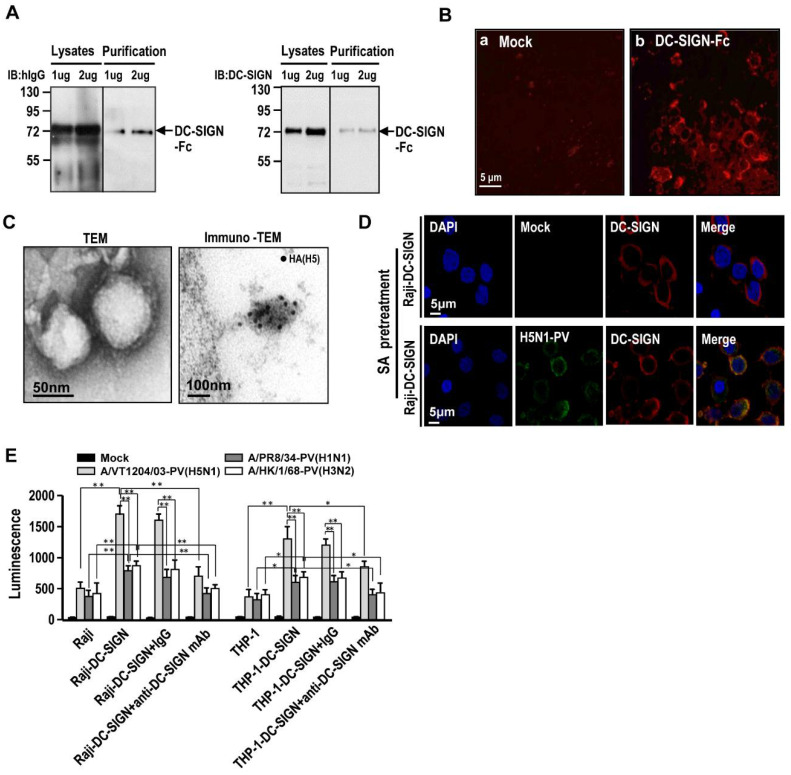
Generation of recombinant DC-SIGN and H5N1 pseduotyped virus. (**A**) The plasmid encoded extracellular carbohydrate interacting domain (ECD) of DC-SIGN with Fc tag was transfected into Freestyle HEK 293F cells and incubated in an orbital shaker incubator for a further 48 h at 37 °C, 120 rpm, and 5% CO2. The supernatants were collected and subjected to further purification by protein A beads. The total lysates and purified recombinant DS-SIGN-ECD-Fc proteins were subjected to immunoblotting using anti-human IgG (the left) or anti-DC-SIGN antibodies (the right) to check protein expression level. (**B**) Mock and pDC-SIGN ECD-Fc transfected cells were subjected to immunofluorescent staining with mouse anti-DC-SIGN antibodies followed by the addition of red fluorescent labeled antibodies against the primary antibodies. The stained cells were observed using a fluorescent microscope. (**C**) Avian H5N1 pseudotyped viruses (PVs) were generated by using co-transfection of pNL-Luc-E-R- vector with pHW1203-HA and pHW1203-NA vectors into HEK293T cells. Cell supernatant containing H5N1-PVs were collected 48 h post-transfection. After purification and concentration by ultracentrifugation, the virions were subjected to transmission electron microscope (TEM), as well as immuno-transmission electron microscope (immuno-TEM) which was stained with anti-HA (H5) antibodies, followed by the addition of gold labeled antibodies against the primary antibodies. (**D**) Sialidase pretreated Raji-DC-SIGN cell were incubated with mock or H5N1-PVs treatment at 4 °C for 2 h for viral binding and subjected to immunofluorescent staining with anti-HA (H5) and anti-DC-SIGN antibodies, followed by the addition of green (for HA) and red (for DC-SIGN) fluorescent labeled antibodies against the primary antibodies and then subjected to Carl Zeiss LSM 700 Confocal Microscope observation. (**E**) Comparison the infectivity among lentivirus pseudotyped with H5N1, H1N1, and H3N2 envelope in Raji and Raji-DC-SIGN cells. Representative results are shown. Quantitative data represent the means ± SD of results from at least three independent experiments (* *p* < 0.05; ** *p* < 0.01).

Next, the human recombinant DC-SIGN (rDC-SIGN) protein was generated using the plasmid expressing DC-SIGN-ECD (ECD; extracellular carbohydrate interacting domain) linked with human IgG Fc transfected to FreeStyle 293-F cell line (Figure S1). The supernatants and lysates were collected and subjected to SDS-PAGE, Coomassie blue staining and immunoblotting. The human recombinant DC-SIGN-Fc protein was expressed successfully (Figure S1). Immunoblotting results demonstrated that rDC-SIGN-Fc proteins were detected from transfected cell lysates and purified supernatants by both anti-DC-SIGN and anti-human IgG antibodies (Figure 3A). Furthermore, the transfected 293-F cells detected DC-SIGN expression via immunofluorescent staining assay (Figure 3B).

Currently, handling of H5N1 AIVs experiment requires a Biosafety Level 3 (BSL-3) facility. For the convenience of this study, a H5N1 pseudotyped virus (PV) was generated by transfecting a HIV-1-defective genome carrying a luciferase gene (pNL-Luc-E-R-) with the vectors encoded HA and NA from A/Vietnam/1203/04 H5N1 into HEK293T cells. The viral supernatants were collected, purified, and subjected to transmission electron microscope (TEM), immuno-electron microscope (immuno-EM), and viral binding assay for structure and functional validation. TEM and immuno-TEM observation demonstrated that the proper structure of the virions and HA5 was detected on the viral envelope (Figure 3C). The ability for DC-SIGN to bind with H5N1-PVs were evaluated via virus binding assay using sialidase pretreated Raji-DC-SIGN cells then incubated with mock or H5N1-PVs treatment. These cells received sialidase treatment could be removed almost α 2–3 SA, the avian influenza virus receptor. Immunofluorescent image revealed that H5N1-PVs were colocalized with DC-SIGN on the surface of Raji-DC-SIGN cells (Figure 3D).

### 2.3. H5N1 Pseudotyped Lentiviruses Utilize DC-SIGN to Enhance Its Infectivity Compared to H1N1 and H3N2 Human Influenza A Viruses

To date, the interaction between DC-SIGN and different types of influenza A virus is not fully understood. We compared the DC-SIGN mediated infectivity of H5N1 AIVs to seasonal influenza A H1N1 and H3N2 viruses using the pseudotyped system. Results indicated that H5N1 (A/Vietnam/1204/03), H3N2 (A/Hong Kong/1/1968), and H1N1 (A/Puerto Rico/8/34) PVs utilize DC-SIGN to enhance infectivity. DC-SIGN expression was noted to significantly facilitate the infectivity in H5N1-PVs compared to H3N2-PVs and H1N1-PVs using Raji/RajiDC-SIGN and THP-1/THP-1-DC-SIGN cells (*p* < 0.01) (Figure 3E). This promotion effect was significantly obstructed by anti-DC-SIGN antibody co-treatment (*p* < 0.05).

### 2.4. N-Glycosylation Sites N27, N39, N181 in Hemagglutinin Proteins of H5N1 AIVs Are Key in Interacting with DC-SIGN

**Figure 4:**
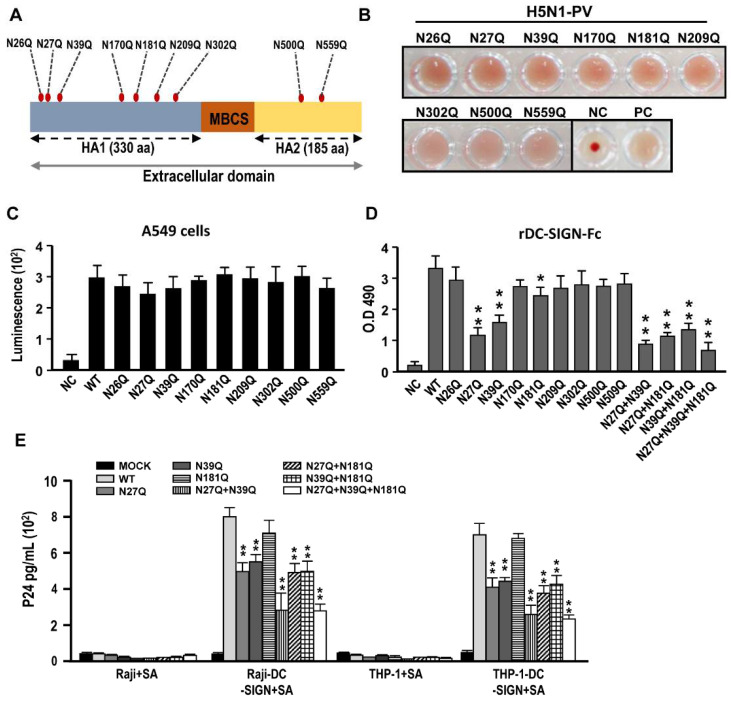
Generation of H5N1-PVs carrying N-glycosylation mutations on HA and evaluation of their infectivity and DC-SIGN interacting abilities. (**A**) The scheme of potential N-glycosylation mutation site on HA are shown. The MBCS indicates “a multi-basic cleavage site.” (**B**) The hemagglutination assay was performed using H5N1-PVs carrying different N-glycosylated mutations and incubated with 0.5% turkey RBC (PC: seasonal H1N1 influenza isolates; NC: PBS). The detail procedure was described in the Materials and Methods. (**C**) Comparison infectivity among the H5N1-PVs carrying different N-glycosylated mutations. (**D**) rDC-SIGN-Fc protein coated ELISA was used to evaluate the binding abilities among the H5N1-PVs carrying different N-glycosylated mutations. (**E**) Sialidase pretreated Raji/Raji-DC-SIGN and THP1-/THP1-DC-SIGN cells were infected with the H5N1-PVs carrying different N-glycosylated mutations. Representative results are shown. Quantitative data represent the means ± SD of results from at least three independent experiments (*p < 0.05; ** *p* < 0.01).

Several N-glycosylation mutants were generated using site-directed mutagenesis to substitute specific Asparagine (N) to Glutamine (Q). A serial N-glycosylation mutation on HA extracellular domain (including HA1 and HA2) of H5N1 viruses were generated including N26Q, N27Q, N39Q, N170Q, N181Q, N209Q, N302Q, N500Q, and N559Q (Figure 4A). These H5N1-PVs with HA N-glycosylation mutations were used to test their hemagglutination and infectious capabilities. Results indicated that the N-glycosylation mutants of the H5N1-PVs displayed hemagglutination capabilities using turkey RBCs (Figure 4B). The infectivity was tested, and the results revealed that these H5N1-PVs with HA N-glycosylation mutations could successfully infect A549 cells with no significant difference (*p* > 0.05) (Figure 4C). Similar results were observed in virus binding assay, indicating that these H5N1-PVs with HA N-glycosylation mutations could bind to sialic acid expressing A549 lung cells (Figure S2) (Table 1).

Additionally, we used rDC-SIGN-Fc, as coating protein in ELISA, to validate the interaction between DC-SIGN and HA N-glycosylation mutants. Results indicated that H5N1-PVs HA with N27Q, N39Q, and N181Q single mutation significantly reduced the binding abilities with rDC-SIGN-Fc (*p* < 0.05). It was further noted that the combination of these N-glycosylation single mutation on HAs had a significantly higher attenuation on their interaction with DC-SIGN (*p* < 0.01) (Figure 4D) (Table 1). Among these combinations, N27Q + N39Q and N27Q + N39Q + N181Q showed additively ameliorated effects when interacting with rDC-SIGN-Fc proteins (Figure 4D). These results were further confirmed by using these HA N-glycosylation mutants of H5N1-PVs incubated with the Raji/Raji-DC-SIGN and THP-1/THP-1-DC-SIGN cells, which were pre-treated with sialidase to majorly cleave and remove α-2,3 SA on the cell surface and subjected to p24 quantification. Results indicated that only N27Q and N39Q N-glycosylation single mutation on HA of H5N1-PVs significantly reduced viral attachment to DC-SIGN expressing cells (*p* < 0.05). Similarly, the combination of these two mutants showed a higher significant and additive attenuated effects in the interaction with DC-SIGN expressing cells (*p* < 0.01) (Figure 4E).

### 2.5. Mutation on N27 and N39 Ameliorate DC-SIGN Mediated H5N1 AIVs Infection in Cis and in Trans

**Figure 5:**
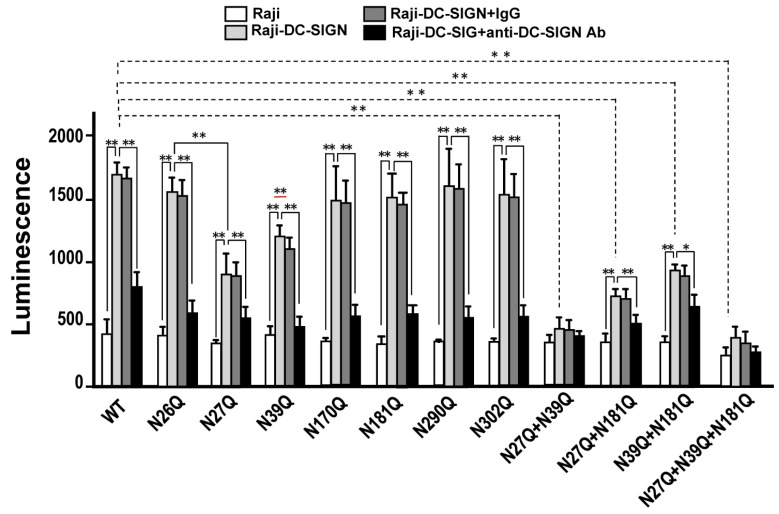
Evaluation of DC-SIGN mediated *cis* infection among H5N1-PVs carrying N-glycosylation mutations. Raji and Raji-DC-SIGN cells were infected with H5N1-PVs carrying different N-glycosylation mutations at 37 °C for 48 h and subjected luminescence activity measurement. WT, wild-type. Representative results are shown. Quantitative data represent the means ± SD of results from at least three independent experiments (* *p* < 0.05; ** *p* < 0.01).

We previously demonstrated that DC-SIGN could mediate H5N1 virus in *cis* and in *trans* [29]. In our current study, we found that N27Q and N39Q on HA of H5N1-PVs significantly attenuated the virus interaction with DC-SIGN expressing cells. Subsequently, we followed up by addressing the effect of these N-glycosylation mutants on H5N1 *cis* and *trans* infection. The H5N1-PVs, which contained N27Q or N39Q and other combination of N-glycosylation mutants on the envelop were subjected to infectivity assay using Raji and Raji-DC-SIGN cells. Results from Figure 5 demonstrated that DC-SIGN expression on Raji cells significantly promoted H5N1-PVs infection (*p* < 0.01) and this promoting effect was significantly obstructed by anti-DC-SIGN mAb co-treatment (*p* < 0.01). Further, we found that DC-SIGN mediated enhancement in viral infectivity could be significantly ameliorated on H5N1-PVs bearing the N27Q or N39Q mutation on the HA (*p* < 0.01). A combination containing these two N-glycosylation mutations resulted in a higher significant reduction in H5N1-PVs infectivity of DC-SIGN expressing Raji cells (*p* < 0.01). We noted that the H5N1-PVs carrying N27Q + N39Q or N27Q + N39Q + N181Q combination displayed the lowest significant DC-SIGN mediated infectivity compared to the wild-type infected control (*p* < 0.01). Combined, these results suggest that mutation on N27 or N39 N-glycosylation site or both sites of HA ameliorate DC-SIGN mediated H5N1 infection in *cis*.

**Figure 6:**
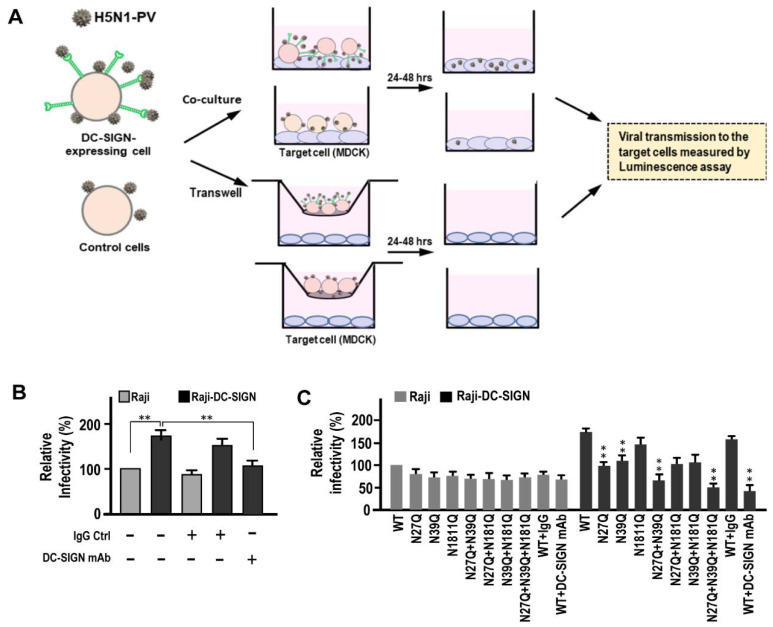
Evaluation of DC-SIGN mediated *trans* infection among H5N1-PVs carrying N-glycosylation mutations. (**A**) The scheme of modified conventional capture assay is demonstrated. (**B**) Raji and Raji-DC-SIGN were used as captured cells. They were incubated with H5N1-PVs at 4 °C for 2 h and then co-cultured with MDCK (target cells) at 37 °C for 24–48 h. After co-culturing, the capture cells were removed via intensive PBS washing (three to five times) and the target MDCK cells were subjected to luminescence analysis. In addition, for detecting the virions budding from *cis* infection, the transwell system was used to monitor those virions released from captured cells further causing MDCK (target cells) infection. The lower channel of infected MDCK cells in the transwell were also subjected to luminescence analysis. Alternatively, some groups were co-treated with IgG control and anti-DC-SIGN monoclonal antibodies. The relative infectivity was measured by using the luminescence values of co-cultured MDCK, normalized with values of MDCK from transwell system. (**C**) The Raji and Raji-DC-SIGN cells (captured cells) were incubated with H5N1-PVs carrying different N-glycosylation mutations on HA at 4 °C for 2 h and then co-cultured with MDCK (target cells) at 37 °C for 24–48 h. After co-culturing, the capture cells were removed via intensive PBS washing (three-five times) and the target MDCK cells were subjected to luminescence analysis. Similarly, the detection of the virions released from *cis* infection of the captured cells was monitored using transwell system mentioned above. Alternatively, some groups were co-treated with IgG control and anti-DC-SIGN monoclonal antibodies. The relative infectivity was measured by using the luminescence values of co-cultured MDCK, normalized with values of MDCK from transwell system. The significant difference was measured by each N-glycosylation mutant compared to WT group. Representative results are shown. Quantitative data represent the means ± SD of results from at least three independent experiments (WT, wild-type) (* *p* < 0.05; ** *p* < 0.01).

Next, we addressed the potential influences of these two N-glycosylation mutations on HA to H5N1 *trans* infection. A modified capture transmission assay was conducted according to our previous work [29]. The scheme is shown in Figure 6A. The Raji and Raji-DC-SIGN cells were used as the capture cells with/without DC-SIGN mAbs co-treatment and incubated with H5N1-PVs then subjected to co-culture with MDCK (as target cells). After co-culturing for 24–48 h, the capture cells were removed by intensive PBS washing (three to five times). The infected MDCK cells, induced by captured cell, were used to measure the luminescence. In addition, the transwell system was used to monitor the potential release virions from *cis* infection of capture cells and then caused infection to the target cells (Figure 6A). Because H5N1-PVs belonged to single cycle infection virus, nono luminescence values were detected from MDCK cells in transwell system. Results indicated that DC-SIGN expression in Raji cells significantly promoted H5N1-PVs infection to the target MDCK cells (*p* < 0.01), while the promoting effect could be significantly blocked by pre-treatment of DC-SIGN mAbs on the capture cells (*p* < 0.05) (Figure 6B). In addition, the H5N1-PVs carrying N27Q or N39Q significantly ameliorated DC-SIGN promoting effect on H5N1 *trans* infection (*p* < 0.05) (Figure 6C). Similar significant and additive reducing effect was observed in H5N1-PVs carrying N27Q + N39Q or N27Q + N39Q + N181Q (*p* < 0.01). However, H5N1-PVs with N181Q mutation did not influence DC-SIGN mediated H5N1 *trans* infection (*p* > 0.05), indicating most attenuated effect was resulted from N27 and N39 N-glycosylation mutation (Figure 6C).

### 2.6. Immature Dendritic Cells (DCs) with Higher DC-SIGN Expressions Display Higher Viral Susceptibility and Cell–Cell Transmission Abilities in Mediating H5N1 Infection

DC-SIGN has been proved to be highly expressed in immature dendritic cells (DCs) (iDCs) compared to mature DCs (mDCs). We wanted to confirm whether the mutation on N27 and N39 ameliorated DC-SIGN mediated H5N1 infection. The monocyte derived DCs were generated according to a previous study [29] and their DC-SIGN expression level was measured by FACS (Figure S3). Higher levels of DC-SIGN expression were detected in iDCs compared to mDCs (Figure S3). The influenza H5N1 NIBRG-14 (H5N1-reverse genetic; -RG) strain was obtained from NHRI. The H5N1-RG was generated via co-transfection of two plasmids encoded HA and NA from A/Vietnam/1203/04 and six plasmids encoded other structure and non-structure proteins from A/PR/8/34. The H5N1-RG strain could be handled in biosafety level-2 (BSL-2) facility.

**Figure 7:**
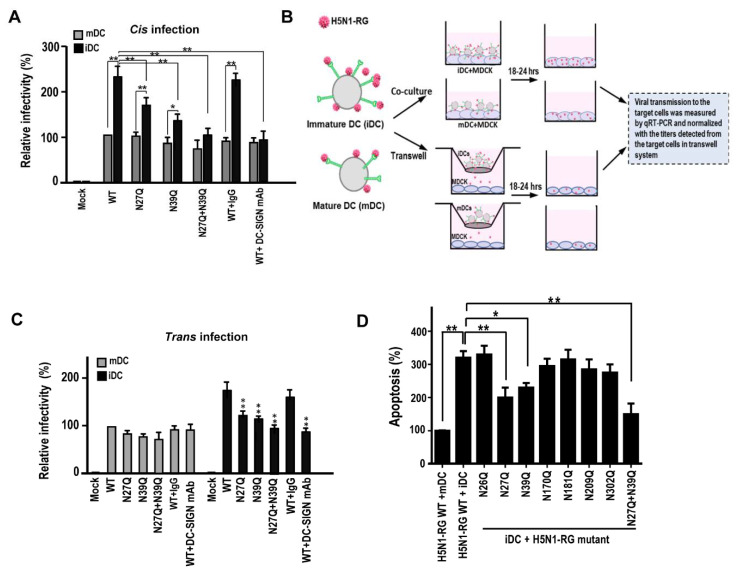
Mutation of 27 and 39 N-glycosylation sites ameliorate DC-SIGN mediated infection in *cis* and in *trans.* (**A**) The monocyte derived immature and mature dendritic cells (iDCs and mDCs) were infected with H5N1-RG viruses carrying different N-glycosylation mutations on HA at 37 °C for 48 h and subjected to qRT-PCR analysis. The relative infectivity was determined by viral copies detected in each group and normalized to wild-type virus infected iDCs. (**B**) The scheme of modified conventional capture assay is demonstrated. (**C**) iDCs and mDCs, as the capture cells, were incubated with H5N1-RG viruses carrying different N-glycosylation mutations on HA at 4 °C for 2 h and subjected to co-culture with MDCK cells (target cells) at 37 °C for 18–24 h. After co-culturing, the virions-captured cells (iDCs and mDCs) were removed via of intensive PBS washing (three to five times) and the MDCK cells were subjected to qRT-PCR analysis. The transwell system was used to monitor the virions budding from *cis* infection (the virions released from captured cells (iDCs and mDCs) are on the upper channel) and further caused MDCK (target cells) (lower channel) infection. The lower channel of MDCK cells receiving the virions from *cis* infection were also subjected to qRT-PCR. The relative infectivity was determined by using the viral copies detected from co-cultured MDCK and normalized with viral copies of MDCK from transwell system. (**D**) The iDCs and mDCs were infected with H5N1-RG wild-type or N-glycosylation mutants and incubated at 37 °C for 48 h. These infected cells were subjected to apoptosis analyses using Annexin V and propidium iodide staining (WT, wild-type). The significant difference was measured by each N-glycosylation mutant compared to WT group. Representative results are shown. Quantitative data represent the means ± SD of results from at least three independent experiments (WT, wild-type) (* *p* < 0.05; ** *p* < 0.01).

H5N1-RG viruses carrying N27Q or N39Q or both mutations on HA were generated using site-directed mutagenesis. To address the correlation between DC-SIGN expression and its effect on viral infectivity, the iDCs and mDCs were infected with H5N1-RG virus. Results from qRT-PCR indicate that significant higher infectivity of H5N1-RG wild-type strain was found in iDCs compared to mDCs (*p* < 0.01) (Figure 7A). As the H5N1-RG virus with N27Q or N39Q on HA significantly ameliorated DC-SIGN mediated *cis* infection in iDCs, similarly, the H5N1-RG carrying N27Q + N39Q on HA additively reduced viral infectivity (*p* < 0.01) (Figure 7A).

Next, we addressed the role of DC-SIGN mediated H5N1 *trans* infection using iDCs and mDCs. The modified conventional capture assay was performed using iDCs or mDCs as the captured cells and incubated with H5N1-RG viruses, and subsequently co-cultured with MDCK (target cells). After co-culturing for 18–24 h, the capture cells were removed via intensive PBS washing (three to five times) and the infected MDCK cells were subjected to qRT-PCR analysis. The transwell system was used to monitor the budding virions released from *cis* infected captured cells and further induce infection of the target cells. The lower channel of MDCK cells receiving the virions from *cis* infection were also subjected to qRT-PCR. The relative infectivity of target cells via DC-SIGN mediated *trans* infection were measured (Figure 7B). Results indicated that significant higher titers of H5N1-RG wild-type strains were detected in iDCs-transmitted MDCK cells compared to mDCs-transmitted MDCK cells (*p* < 0.01) (Figure 7C). Furthermore, significant lower titers of H5N1-RG viruses with N27Q or N39Q mutations on HA were detected in iDCs-transmitted MDCK cells (*p* < 0.01). Similarly, H5N1-RG viruses with both N-glycosylated mutations showed additive attenuating effects on DC-SIGN mediated *trans* infection by iDCs (Figure 7C). In addition, we noted that significant higher virus induced apoptosis by H5N1-RG was found in iDCs than mDCs (*p* < 0.01). H5N1-RG carrying N27Q or N39Q or both mutations significantly reduced H5N1 induced apoptosis in iDCs (*p* < 0.05) (Figure 7D).

## 3. Discussion

The binding of influenza A viruses to cells are not restricted to the recognition of sialic acids by the receptor binding site (RBS) of HA. It has been reported that influenza A viruses can bind to lectin receptors, suggesting that lectin-HA interaction might be involved in virus attachment and subsequent viral entry [37,38]. We previously addressed the role of DC-SIGN in H5N1 AIVs infection and demonstrated that DC-SIGN can serve as an attachment molecule to facilitate H5N1 infection [29]. In this study, we extended the previous findings to further identify the important DC-SIGN interaction with the N-liked glycosylation sites in HA of H5N1 AIVs. Our results indicate that N27 and N39 are two essential N-linked glycosylation sites in HA protein which are involved in DC-SIGN interaction in H5N1 AIVs. Mutations on these two N-glycosylation sites significantly reduced HA binding to DC-SIGN, and ameliorated DC-SIGN positive regulatory effects on H5N1 infection in *cis* and in *trans*. This is the first study to address the optimal N-glycosylation sites in HA of H5N1 AIVs.

DC-SIGN is a transmembrane C-type lectin receptor with a long extracellular neck region and a carbohydrate recognition domain (CRD) [21]. DC-SIGN is known to have high affinity to N-linked high-mannose oligosaccharides and branched fucosylated structures [39]. The N-linked glycans are found commonly on viruses, bacteria, and fungi [21,39]. Our results suggest that almost all human H5N1 isolates from clade 0 to clade 7, during 2004–2020, contain the same pattern and numbers of N-linked glycosylation sites on the HA protein (Figure 1 and Figure 2). Previous studies have shown that N-linked glycosylation on HA of human influenza A viruses could assist to hide viral antigen to reduce immune detection and attack [21,40]. Under circulation and evolution, the pattern and numbers of N-glycosylation sites have been altered and modified in human influenza A viruses (such as H1N1 and H3N2) as reported in previous studies [7,41]. However, these changes were not found in the human H5N1 isolates used in this study. Accordingly, we suggest that H5N1 has not evolved in humans for a long time and this N-glycosylation pattern found in H5N1 might assist the viruses to escape immune attack, and additionally, to interact with lectins such as DC-SIGN to enhance viral infection.

Currently, H5N1 AIVs has reported high mortality in human infection and is listed as a bio-safety level (BSL)-3 pathogen. Owing to lack of such facility, the pesudotyped virus system (belonged to BSL-2 pathogen) was used in this study. The H5N1-PVs were generated using a vector expressing lentivirus core structure combined with vectors expressing HA5 and NA1 proteins (Figure 3). The hemagglutination and infectivity of H5N1 pesudotyped viruses were validated and immuno-TEM was utilized to further prove the similarity of the virological function with avian H5N1 viruses (Figure 3). A similar strategy has also been used in several previous publications [29,42,43], suggesting that the lentivirus particles with the H5N1 envelope is a suitable tool allowing for handling in a common virological laboratory.

Firstly, we screened for the important N-glycosylation sites expressed on HA of H5N1 virus by rDC-SIGN protein coated ELISA, to interact with H5N1-PVs bearing different mutagenetic N-liked glycosylation sites on HA. A similar strategy was used in a previous study by Hong et al. [44], who used rDC-SIGN proteins to screen HIV-1 viruses carrying different N-glycosylation mutations on HIV-1 gp120 envelope. In this study, we found that N27, N39, and N181 on HA could be critical in interacting with rDC-SIGN proteins using ELISA assay. These three N-glycosylation sites were located on the side and top region of HA1 structure. Further, we used monocyte derived iDCs and mDCs to confirm these findings and found that only N27Q and N39Q mutations significantly reduced DC-SIGN mediated H5N1 virus infection. However, this amelioration effect was not observed in H5N1 virus carrying N181Q mutation. We suggest that the interaction between HA and DC-SIGN might be influenced by the quality and purity of rDC-SIGN proteins in the ELISA. In addition, the native form of DC-SIGN is a tetrameric transmembrane protein. Accordingly, we propose that using DC-SIGN expressing cells are better and mimic the real-life situation compared to using artificial rDC-SIGN proteins. Nevertheless, several studies still suggest that rDC-SIGN is a good candidate for fast screening of important DC-SIGN interactive N-linked glycosylation sites [26,44].

Currently, the detailed interaction and binding mechanism between DC-SIGN and distribution or distance of N-linked glycan sites remains controversial. DC-SIGN is known to bind to two classes of carbohydrate structures: N-linked high mannose oligosaccharides (such as Man9GlcNAc2) and branched, fucosylated oligosaccharides [45,46]. High-mannose glycans are abundantly detected on many types of enveloped viruses [47].

Menon et al., reported that crystal structures of DC-SIGN CRD complex with oligosaccharide ligands revealed that extensive ligand-binding site in DC-SIGN results in higher-affinity of one-to-one interactions with specific glycans (e.g., DC-SIGN CRD forms a one-to-one complex with a high-mannose oligosaccharide), although engagement of such ligands is more geometrically constrained [48]. The oligosaccharide is known to only be accommodated in a single orientation, thus this specificity places spatial constraints for DC-SIGN interaction with high-mannose glycans on pathogen surfaces [49]. Other studies also indicate that DC-SING with flexibility to bind to the ligands, allows multiple CRDs in a tetramer to interact with more complex but more sparsely spaced glycan ligands on a membrane [48,49]. In addition, Medina et al. noted that the N-glycosylation of the influenza hemagglutinin plays an important role in the life cycle of influenza virus and plays a key role on its antigenic fitness. Indeed, oligosaccharides attached to the globular head of HA have been shown to modulate virus antigenic properties and its receptor binding [50]. Our results demonstrated that most single mutations on N-linked glycosylation sites on HA5 did not influence the binding between DC-SIGN and HA (H5), whereas only three mutations (N27Q, N39Q, and N181Q) reduced this interaction; suggesting that the DC-SIGN CRD may interact minimally with a N-glycosylation with high-mannose oligosaccharides expression on HA protein. When it came to N181, which was located on the top side of HA structure, this interaction might only be displayed with a high dose of recombinant monomeric DC-SIGN protein instead of tetrameric native structure. We suggested that this interaction might be non-specific or weak which led to no significant interaction with tetrameric form of DC-SIGN expressing cells. The details for this phenomenon require further investigation.

**Figure 8:**
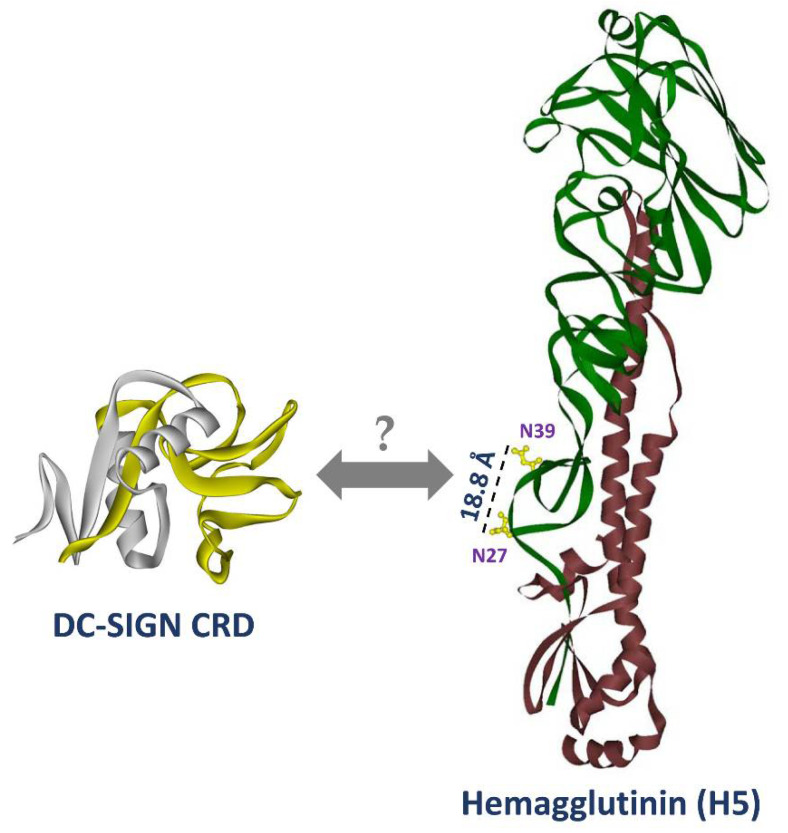
The scheme of the structure DC-SIGN carbohydrate recognition domain (CRD) lectin-interacting domain and two essential N-glycosylation sites shown on H5N1 hemagglutinin protein. The lectin-interacting domain of DC-SIGN CRD and N27 and N39 N-glycosylation sites on H5N1 hemagglutinin structure is demonstrated. The distance between N27 and N39 was measured using DS Viewer program. The yellow labeling structure in DC-SIGN CRD (PDB 1XAR) indicates lectin-binding domain according to the reference published elsewhere [52]. The green and red labeling structures of hemagglutinin indicate HA1 and HA2, respectively (PDB 2FK0). The DC-SIGN CRD and H5 structures of this figure were manually superimposed instead of showing the co-crystal structure results.

We also noted that combination of two N-glycosylation mutations on HA additively ameliorated DC-SIGN binding to HA5, and reduced DC-SIGN-mediated promoting effect on H5N1 *cis* and *trans* infection. Previously, Zhang et al. reported that soluble DC-SIGN bound to dengue virus, suggesting that the CRD monomer can also bind to two N-glycosylation sites spaced 18 Å apart on adjacent envelope dimers on the virion surface using cryo-electron microscopy analysis, proposing that the CRD has flexibility in the interaction with two N-glycosylation sites [51]. The two carbohydrate moieties bind to a single CRD monomer, which might be a result of the elongated oligosaccharide binding valley present in the CRD of DC-SIGN [42,45]. Another study noted that the distance between N-glycosylation sites was approximately 19 to 23 Å, thus fulfilling the minimal spatial requirement for at least two N-glycosylation sites for binding to a single DC-SIGN CRD [44]. Our results estimated that the distance between N27 and N39 was around 18.6 Å (Figure 8). This distance is similar to results found in dengue virus. Accordingly, we suggest that the distance (18.6 Å) between two identified N-linked glycosylation sites in this study still fit the minimal requirement regarding DC-SIGN binding.

There were some limitations to this study. We simply expressed the recombinant extracellular domain of DC-SIGN for the screening of the N-linked glycosylation sites. The purity of rDC-SIGN proteins may influence their interaction with interactive proteins, especially those weak interactive proteins. Most of the results in this study used the H5N1 PVs or H5N1-RG. Although these viruses were reported as representative candidates for studying the function of HA or NA, the real H5N1 AIVs are still necessary to validate and confirm the findings in this study. Because it might be worth considering the glycomics of the different cell lines and how glycosylation of the H5N1 PV particle released from HEK293T cells may not be the same as glycosylation of the HA of the virus grown in other cellular substrates or in natural infection, and so the DC-SIGN recognition may be related to the system used to generate the virus. Further, the fine-tune mechanism regarding how DC-SIGN regulates these H5N1 mutants containing different N-glycosylation mutations was not performed. The detail mechanism including DC-SIGN mediated endocytosis, as well as the downstream signaling pathway, are worthy of further investigating.

In this study, we generated recombinant DC-SIGN proteins which were used to establish ELISA based screening assay to map essential N-linked glycosylation sites on HA protein of H5N1 viruses. Our data demonstrated that N27 and N39 are two essential N-glycosylation sites, which are majorly involved in the binding to the DC-SIGN. Mutation on these two N-glycosylation sites on HA significantly ameliorated DC-SIGN mediated H5N1 infection in *cis* and in *trans*. Further investigation is demanded to understand the DC-SIGN regulatory mechanism on H5N1 AIVs and even other subtypes of influenza A virus infection.

## 4. Materials and Methods

### 4.1. Ethics Statement

Written informed consent was elicited from all study participants. Approval was applied for and received from the Institutional Ethics Committee of the Kaohsiung Medical University, Taiwan. All procedures were conducted according to committee regulations.

### 4.2. Cell

HEK293T (human kidney), Madin-Darby canine kidney (MDCK), Raji B, Raji-DC-SIGN (a stable B-THP-1 clone expressing DC-SIGN molecules), THP-1 (monocyte), and THP-1-DC-SIGN (a stable THP-1 clone expressing DC-SIGN molecules) were used in this study. HEK293T and MDCK cells were cultured in Dulbecco’s modified Eagle’s medium (DMEM; Gibco-BRL, Rockville, MD, USA) supplemented with 10% heat-inactivated fetal bovine serum (HyClone™ Characterized Fetal Bovine Serum, U.S.), penicillin (100 U/mL), streptomycin (100 μg/mL), non-essential amino acids (0.1 mM), and L-glutamine (2 mM) (GIBCO-BRL). Raji, Raji-DC-SIGN, THP-1, and THP-1-DC-SIGN cells were cultured in RPMI 1640 medium (GIBCO-BRL) supplemented with 10% heat-inactivated fetal calf serum, penicillin (100 U/mL), and streptomycin (100 μg/mL). For the B-THP-1/DC-SIGN cell line, 50 μg/mL of neomycin (Sigma-Aldrich) was added to the medium.

### 4.3. H5N1 Pseudotype and Reverse-Genetic Virus Preparation

The full-length of HA and NA from A/Vietnam 1203/04 were amplified using RT-PCR with the primers containing the universal conserved non-coding regions of influenza A virus (AGCAAAAG CAGG or AGTAGAAACAAGG). The details are described elsewhere [53]. The primers used in the PCR reaction contained segment specific sequences and *Bsm*BI or *Bsa*I restriction site sequences at their end. After digestion of the PCR products with *Bsm*BI or *Bsa*I, the fragments of HA5 and NA1 were cloned into the vector, pHW2000. The H5N1 pseudo-type viruses (PVs) were generated using co-transfection of pNL-Luc-E-R- vector with pHW1203-HA and pHW1203-NA vectors into HEK293T cells. Cell supernatant containing H5N1-PVs were collected 48 h post-transfection and purified through a 0.45 μm filter. Supernatant was concentrated by ultracentrifugation at 25,000 rpm for 2.5 h to obtain high concentration of H5N1 PV particles. Regarding H5N1 reverse-genetic virus, the detailed protocol is described elsewhere [53]. Briefly, the two plasmids encoded HA and NA of A/Vietnam/1203/04 strain and the other six plasmids encoded structure and non-structure proteins of A/Puerto Rico/8/34 stain were co-transfected into HEK293T and incubated at 37 °C for 48 h. The viral supernatants were collected and subjected to propagation in Vero or MDCK. The H5N1-RG was used as vaccine candidate and could be handled in biosafety level 2 facility (BSL-2) [54].

### 4.4. N-Linked Glycosylation Site Prediction

Predictions of N-linked glycosylation on H5N1 hemagglutinin protein were performed using the NetNGlyc 1.0 Server (http://www.cbs.dtu.dk/services/NetNGlyc/). The full length of hemagglutinin amino acid sequence from the influenza A H5N1 (A/Vietnam/1203/04 strain; accession no. ABW90135) and other human H5N1 isolates from 2004–2020 searched and downloaded from NBCI Influenza Resource (https://www.ncbi.nlm.nih.gov/genomes/FLU/Database/nph-select.cgi?go=database). There was a total of 274 human H5N1 isolates obtained and most H5N1 isolates were found during 2003–2015. After collapsing identical sequence, all the HA sequences were uploaded to the website and total N-linked glycosylation sites were predicted following the Asn-X-Ser/Thr rule. The 3D hemagglutinin structure file was downloaded from the Protein Data Bank (file name: 2FK0). N-linked glycosylation sites were labeled on the structure shown via the DS ViewerPro 5.0 program.

### 4.5. Mutagenesis of N-Linked Glycosylation Site of HA (H5) Protein

To mutate the N-linked glycosylation sites from Asn (N) to Gln (Q), the QuikChange II XL site-directed mutagenesis kit (Stratagene, La Jolla, CA, USA) was used. The detailed protocols were published elsewhere [55]. Forward and reverse primers were designed for the predicted N–linked glycosylation sites. For this study, overlapping PCR was done on pHW1203-HA, following by treatment with *Dpn*I restriction endonuclease digestion for 2 h at 37 °C. The pHW1203-HA with different N-linked glycosylation mutations including N26Q, N27Q, N39Q, N170Q, N181Q, N209Q, N302Q, N500Q, N599Q and other combination of mutations were generated. All of the plasmids were confirmed by sequencing using a dye terminator cycle sequencing core kit on a DNA sequencer (Applied Biosystems, Foster City, CA, USA).

### 4.6. Immuno-Electron Microscopy (Immuno-EM)

The detailed protocol of immuno-EM was described in previous studies [56,57]. Briefly, pNL-Luc-E-R-, pHW1203-HA, and pHW1203-NA vectors were transfected to HEK293T (2:1:1 ratio) and incubated at 37 °C for 48 h. The viral supernatants and cell pellets were collected for immuno-EM staining. Supernatants were filtered (0.45-mm mesh), placed on 20% sucrose, and subsequently ultracentrifuged at 100,000× *g* for 2 h; cells were resuspended in 50 μL PBS. Viral droplets were placed on grids and fixed with 0.5% glutaraldehyde and 4% paraformaldehyde for 10 min. Grids were washed three times with PBS and blocked with 1% fish gelatin and held at room temperature for 30 min. Immunolabeling was performed using primary mouse anti-HA (H5) monoclonal antibodies and incubated overnight at 4 °C. After three PBS washes, grids were incubated with secondary antibodies labeled with 10-nm gold particles (goat anti-mouse IgG polyclonal Ab) and held for 1 h at 37 °C. After three additional PBS washes, grids were stained with 4% uranyl acetate and lead citrate. Electron micrographs were obtained using a Hitachi H-7000 Transmission Electron Microscope.

### 4.7. ELISA

The pcDNA3/hIgG1.Fc(mut)-DC-SIGN.ECD vector was kindly offered by Dr. Jason C. Huang at National Yang Ming University. This plasmid expressing recombinant DC-SIGN extracellular domain was produced in 200 mL suspension cultures of the FreeStyle™ 293-F cells in FreeStyle 293 Expression Medium (Gibco). The detailed protocol is described elsewhere [58]. Equal amounts of purified recombinant DC-SIGN proteins (1 μg/well) were coated on 96 well plate and then blocked by 5% bovine serum albumin [BSA]. Next, the H5N1-PVs wild-type or mutants, with different N-linked glycosylation mutations, were incubated with recombinant DC-SIGN protein coated ELISA to test their binding abilities to DC-SIGN for 2 h at 37 °C. After three washing with PBST, the anti-p24 monoclonal antibody (1:1000) was used to detect the binding H5N1-PVs.

### 4.8. Immunofluorescent Staining

For confirmation of expression of DC-SIGN, the pcDNA3/hIgG1.Fc(mut)-DC-SIGN.ECD vectors were transfected to 293-F cells and incubated at 37 °C for 48 h. The cells were subjected to immunostaining with mouse anti-DC-SIGN monoclonal antibodies (R&D, Cat. No. MAB161) (1:1000) and incubated at room temperature for 1 h. After three washes with PBS, the cells were strained with goat anti-mouse-IgG conjugated Alexa555 (Abcam, Cat. No. ab150118) (1:1000). After repeating the PBS wash, the stained cells were observed using a Carl Zeiss LSM 700 Laser Scanning Confocal Microscope. For observation of the H5N1-PVs binding to DC-SIGN expressing on the cells, Raji and Raji-DC-SIGN cells were pre-treated with sialidase (0.25 U/mL) for 1–3 h and these cells were incubated with H5N1-PVs at 4 °C for 2 h. After washing with PBS, these cells were fixed with 4% paraformaldehyde fixation and blocked with 1% BSA. The cells were then stained with rabbit anti-HA5 polyclonal antibodies (in house preparation) (1:1000) and mouse anti-DC-SIGN monoclonal antibodies (R&D, Cat. No. MAB161) (1:1000), followed by the addition of fluorescently labeled antibodies against the primary antibodies. After mounting, stained cells were observed using a Carl Zeiss LSM 700 Laser Scanning Confocal Microscope.

### 4.9. Cis Infectivity Assay

The equal amounts of H5N1 pseudotyped virus particles (quantification with a Coulter HIV-1 p24 antigen assay (Beckman Coulter)) were collected and incubated with Raji, Raji-DC-SIGN, THP-1, THP-1-DC-SIGN cells, as well as immature DC (iDC) and mature DC (mDC). Luciferase activity was measured in cell lysate after 48 h of incubation at 37 °C. For a DC-SIGN-enhanced infectivity assay, a 5 × 10^5^ susceptible cells mentioned above were seeded into 48-well plates prior to incubation with H5N1 pseudotyped or H5N1-RG virus particles at 37 °C for 2 h. Alternatively, some of these cells were pretreated with anti-DC-SIGN monoclonal antibodies (10 μg/mL-1; R&D System, catalog no. MAB161). After incubation, virus-bound cells were washed 3 times with PBS and harvested. Quantities of pseudotyped H5N1 or RG strain particles were determined by real-time reverse transcription (RT)-PCR (for detecting H5N1 gene expression) [59] and luciferase activity (for the pseudotyped virus particles) [26,29].

### 4.10. Trans Infectivity Assay

DC-SIGN-mediated virus transfer efficiency was assessed by capture assay as described previously [26,31]. Briefly, the 5 × 10^5^ captured cells (Raji, Raji-DC-SIGN, THP-1, THP-1-DC-SIGN, iDC and mDC) were incubated with H5N1-PVs (50 ng p24 equivalent) or 100 uL of H5N1-RG viruses (10^5^ viral RNA copies/mL) at 4 °C for 2 h for virus binding, then washed with ice-cold PBS three times before being added to the target MDCK cells. Target cells were harvested after co-culturing for 18–24 h or 24–48 h. H5N1 viral RNA quantification was performed using qRT-PCR. Quantification of the H5N1-PVs titers was achieved using a luciferase reporter assay kit (Promega, Madison, WI, USA) and a luminometer (PerkinElmer, Waltham, MA, USA). For controlling the budding virions from *cis* infected captured cells and then caused additional infection to the target cells. The transwell system (Corning Costar, Amsterdam, The Netherlands), which separated virions captured cells (upper channel) and target cell (lower channel) was used to monitor this phenomenon. The relative infectivity of the virus transmitted target cells was determined by the viral titers measured in co-cultured target cells, normalized with the viral titers measured in the target cells from the transwell system.

### 4.11. qRT-PCR

Different cells or cell lines used in this study were infected with H5N1-RG viruses. The infected cell lysates were collected and subjected to RNA extraction using TOOLS EsayPrep total RNA kit (Cat. No. DPT-BD19, BIOTOOLS Co., Ltd., Taipei, Taiwan). After RNA extraction, reverse transcription was performed on the isolated RNA using the ToolsQuant II Fast RT kit (Cat. No. KRT-BA06, BIOTOOLS Co., Ltd., Taipei, Taiwan). The cDNA was further subjected to quantitative real-time PCR using the TOOLS SuperFast SYBR qPCR reagent (with ROX dye) (Cat.No.FPT-BB07, BIOTOOLS Co., Ltd., Taipei, Taiwan) with ABI 7000 real-time PCR machine. The primers for detection of specific HA gene of H5N1 virus were listed in previous studies [29,59]. H5N1 viral quantities were calculated by interpolation from a standard curve generated by the parallel running of serial dilutions of known quantities of the H5 segments of cloned plasmids.

### 4.12. Generation of Monocyte-Derived Dendritic Cells (MDDCs)

For preparation of monocyte-derived dendritic cells, the protocol used in this study was published elsewhere [26]. Briefly, the peripheral blood mononuclear cells (PBMCs) were isolated from whole blood using Ficoll-Paque Plus (GE Healthcare Bio-Sciences AB, Umea, Sweden) reagent. Further, the monocytes were extracted from PBMCs with anti-CD14 microbeads (Miltenyi Biotec, Bergisch Gladbach, Germany) via standard density gradient centrifugation. Isolated human monocytes were cultivated in RPMI 1640 medium supplemented with 10% fetal calf serum, 800 U/mL human granulocyte-macrophage colony-stimulating factor (R&D Systems, Minneapolis, MN, USA), and 500 U/mL human interleukin-4 (R&D) for 6 days to trigger differentiation into immature DCs (iDCs). iDCs were confirmed with CD1a, CD40, CD54, HLA-DR, CD80, CD83, CD86, and DC-LAMP cell markers by flow cytometry and morphological characteristics. To induce the transformation of iDCs into mature DCs (mDCs), 0.1 ug/mL lipopolysaccharide (LPS) was added to the RPMI 1640 medium for 1.5 days. mDCs were also confirmed using the above-mentioned cell markers and morphological characteristics. The DC-SIGN expression levels between iDCs and mDCs were measured by flowcytometry.

### 4.13. Apoptosis Assays

H5N1-RG induced cell apoptosis assays were performed using Annexin V and propidium iodide staining [60]. The detail protocol was described elsewhere. Briefly, iDCs or mDCs were infected with H5N1-RG which carried different N-linked glycosylation mutations. The infected cells were stained with FITC-conjugated Annexin V (Calbiochem) according to manufacturer’s recommendations. Annexin V-FITC was diluted in the manufacturer’s Hepes-buffer (containing 2.5 mM CaCl2), added to the cultures, and incubated for 15 min at room temperature. Further, the cells were then fixed with 1% paraformaldehyde, permeabilized with 0.1% Triton, and then incubated with 2.5 ug/mL propidium iodide (PI) in PBS containing 1.2 uL/mL DNAse-free RNAse. The cells stained by Annexin V and PI were subjected to FACS analysis and observed under fluorescence microscope (Zeiss).

### 4.14. Hemagglutination Assay (HA)

The hemagglutination activity of H5N1 pseudtotyped viruses (PVs) carrying different N-glycosylation mutations was performed using 0.5% turkey RBCs as described previously [61]. Briefly, the erythrocytes from turkey were suspended in phosphate-buffered saline (PBS), pH 7.4. In a U-bottomed 96-well plate, 50 μL of 10-fold dilution of purified H5N1-PVs and 50 μL of turkey RBCs were added and gently mixed. The reaction mixture was incubated at room temperature for 45 min. The virus caused hemagglutination was observed.

### 4.15. Statistical Analyses

All experiments were performed at least three times each. Statistical analyses were preformed using GraphPad Prism software. Statistical significance (*p* < 0.05) was calculated using unpaired Student’s *t*-tests.

## Figures and Tables

**Table 1:** Binding of wild-type and N-glycosylation mutants in HA of H5N1.

Mutation(s) in H5N1 Construct from Strain			Binding of HA5 to:	
			DC-SIGN	SA Receptor
None (wild-type)			+	+++
N26Q			+	+++
N27Q			+	++
N39Q			+	+++
N170Q			+	+++
N181Q			+	+++
N209Q			+	+++
N302Q			+	+++
N500Q			+	+++
N559Q			+	+++
N26Q	N27Q		+	++
N26Q	N181Q		+	+++
N27Q	N181Q		+	+++
N26Q	N27Q	N181Q	+	++

Levels of binding are indicated as follows. DC-SIGN: +, 50 to 100% of wild-type binding; +, <50% of wild-type binding. SA: +++, 80 to 100% of wild-type binding; ++, 50 to 80% of wild-type binding.

## Data Availability

The data presented in this study is contained within the article.

## Supplementary Materials

Supplementary Materials can be found at https://www.mdpi.com/1422-0067/22/2/743/s1.

## References

[1] Claas E.C., Osterhaus A.D., Van Beek R., De Jong J.C., Rimmelzwaan G.F.A., Senne D., Krauss S., Shortridge K.F., Webster R.G. (1998). Human influenza A H5N1 virus related to a highly pathogenic avian influenza virus. Lancet.

[2] Chowdhury S., Hossain M.E., Ghosh P.K., Ghosh S., Hossain M.B., Beard C., Rahman M., Rahman M.Z. (2019). The Pattern of Highly Pathogenic Avian Influenza H5N1 Outbreaks in South Asia. Trop. Med. Infect. Dis..

[3] Peiris J.S.M., De Jong M.D., Guan Y. (2007). Avian Influenza Virus (H5N1): A Threat to Human Health. Clin. Microbiol. Rev..

[4] WHO/OIE/FAO (2012). Continued evolution of highly pathogenic avian influenza A (H5N1): Updated nomenclature. Influenza Other. Respir. Virus..

[5] Xu L., Bao L., Yuan J., Li F., Lv Q., Deng W., Xu Y., Yao Y., Yu P., Chen H. (2013). Antigenicity and transmissibility of a novel clade 2.3.2.1 avian influenza H5N1 virus. J. Gen. Virol..

[6] WHO (2020). Cumulative Number of Confirmed Human Cases for Avian Influenza A(H5N1) Reported to WHO. https://www.who.int/influenza/human_animal_interface/H5N1_cumulative_table_archives/en/.

[7] Wang S.-F., Lee Y.-M., Chan Y.-J., Liu H.-F., Yen Y.-F., Liu W.-T., Huang J.C., Chen Y.-M.A. (2009). Influenza A virus in Taiwan, 1980–2006: Phylogenetic and antigenic characteristics of the hemagglutinin gene. J. Med. Virol..

[8] Palese P. (2007). Orthomyxoviridae: The Viruses and their Replication. Fields Virol..

[9] Bouvier N.M., Palese P. (2008). The biology of influenza viruses. Vaccine.

[10] Shinya K., Ebina M., Yamada S., Ono M., Kasai N., Kawaoka Y. (2006). Avian flu: Influenza virus receptors in the human airway. Nature.

[11] Komar N., Olsen B. (2008). Avian Influenza Virus (H5N1) Mortality Surveillance. Emerg. Infect. Dis..

[12] Oslund K.L., Baumgarth N. (2011). Influenza-induced innate immunity: Regulators of viral replication, respiratory tract pathology & adaptive immunity. Futur. Virol..

[13] Zhang Z., Zhang J., Huang K., Li K.-S., Yuen K.-Y., Guan Y., Chen H., Ng W.F. (2009). Systemic infection of avian influenza A virus H5N1 subtype in humans. Hum. Pathol..

[14] Korteweg C., Gu J. (2008). Pathology, Molecular Biology, and Pathogenesis of Avian Influenza A (H5N1) Infection in Humans. Am. J. Pathol..

[15] Wang S.-F., Chen K.-H., Thitithanyanont A., Yao L., Lee Y.-M., Chan Y.-J., Liu S.-J., Chong P., Liu W.T., Huang J.C. (2009). Generating and characterizing monoclonal and polyclonal antibodies against avian H5N1 hemagglutinin protein. Biochem. Biophys. Res. Commun..

[16] Mason C.P., Tarr A. (2015). Human Lectins and Their Roles in Viral Infections. Molecules.

[17] Chen Y.-J., Wang S.-F., Weng I.-C., Hong M.-H., Lo T.-H., Jan J.-T., Hsu L.-C., Chen H.-Y., Liu F.-T. (2018). Galectin-3 Enhances Avian H5N1 Influenza A Virus–Induced Pulmonary Inflammation by Promoting NLRP3 Inflammasome Activation. Am. J. Pathol..

[18] Wang W.-H., Yeh C.-S., Lin C.-Y., Yuan R.-Y., Urbina A.N., Lu P.-L., Chen Y.-H., Chen Y.-M.A., Liu F.-T., Wang S.-F. (2020). Amino Acid Deletions in p6Gag Domain of HIV-1 CRF07_BC Ameliorate Galectin-3 Mediated Enhancement in Viral Budding. Int. J. Mol. Sci..

[19] Li F.-Y., Wang S.-F., Bernardes E.S., Liu F.-T. (2020). Galectins in Host Defense against Microbial Infections. Cannabinoids Neuropsychiatr. Disord..

[20] Wang W.-H., Lin C.-Y., Chang M.R., Urbina A.N., Assavalapsakul W., Thitithanyanont A., Chen Y.-H., Liu F.-T., Wang S.-F. (2020). The role of galectins in virus infection—A systemic literature review. J. Microbiol. Immunol. Infect..

[21] Van Kooyk Y., Geijtenbeek T.B.H. (2003). DC-SIGN: Escape mechanism for pathogens. Nat. Rev. Immunol..

[22] Cunningham A.L., Harman A.N., Donaghy H. (2007). DC-SIGN ’AIDS’ HIV immune evasion and infection. Nat. Immunol..

[23] Poehlmann S., Zhang J., Baribaud F., Chen Z., Leslie G.J., Lin G., Granelli-Piperno A., Doms R.W., Rice C.M., McKeating J.A. (2003). Hepatitis C Virus Glycoproteins Interact with DC-SIGN and DC-SIGNR. J. Virol..

[24] Simmons G., Reeves J.D., Grogan C.C., Vandenberghe L.H., Baribaud F., Whitbeck J.C., Burke E., Buchmeier M.J., Soilleux E.J., Riley J.L. (2003). DC-SIGN and DC-SIGNR Bind Ebola Glycoproteins and Enhance Infection of Macrophages and Endothelial Cells. Virology.

[25] Tassaneetrithep B., Burgess T.H., Granelli-Piperno A., Trumpfheller C., Finke J., Sun W., Eller M.A., Pattanapanyasat K., Sarasombath S., Birx D.L. (2003). DC-SIGN (CD209) Mediates Dengue Virus Infection of Human Dendritic Cells. J. Exp. Med..

[26] Shih Y.-P., Chen C.-Y., Liu S.-J., Chen K.-H., Lee Y.-M., Chao Y.-C., Chen Y.-M.A. (2006). Identifying Epitopes Responsible for Neutralizing Antibody and DC-SIGN Binding on the Spike Glycoprotein of the Severe Acute Respiratory Syndrome Coronavirus. J. Virol..

[27] Hillaire M.L.B., Nieuwkoop N.J., Boon A.C.M., De Mutsert G., Trierum S.E.V.-V., Fouchier R.A.M., Osterhaus A.D.M.E., Rimmelzwaan G.F. (2013). Binding of DC-SIGN to the Hemagglutinin of Influenza A Viruses Supports Virus Replication in DC-SIGN Expressing Cells. PLoS ONE.

[28] Londrigan S.L., Turville S.G., Tate M.D., Deng Y.-M., Brooks A.G., Reading P.C. (2010). N-Linked Glycosylation Facilitates Sialic Acid-Independent Attachment and Entry of Influenza A Viruses into Cells Expressing DC-SIGN or L-SIGN. J. Virol..

[29] Wang S.-F., Huang J.C., Lee Y.-M., Liu S.-J., Chan Y.-J., Chau Y.-P., Chong P., Chen Y.-M.A. (2008). DC-SIGN mediates avian H5N1 influenza virus infection in cis and in trans. Biochem. Biophys. Res. Commun..

[30] Hong P., Ninonuevo M.R., Lee B., Lebrilla C., Bode L. (2009). Human milk oligosaccharides reduce HIV-1-gp120 binding to dendritic cell-specific ICAM3-grabbing non-integrin (DC-SIGN). Br. J. Nutr..

[31] Geijtenbeek T.B., Kwon D.S., Torensma R., Van Vliet S.J., Van Duijnhoven G.C., Middel J., Cornelissen I.L., Nottet H.S., KewalRamani V.N., Littman D.R. (2000). DC-SIGN, a Dendritic Cell–Specific HIV-1-Binding Protein that Enhances trans-Infection of T Cells. Cell.

[32] Wang S.-F., Su M.-W., Tseng S.-P., Li M.-C., Tsao C.-H., Huang S.-W., Chu W.-C., Liu W.-T., Chen Y.-M.A., Huang J.C. (2016). Analysis of codon usage preference in hemagglutinin genes of the swine-origin influenza A (H1N1) virus. J. Microbiol. Immunol. Infect..

[33] Kim P., Jang Y.H., Kwon S.B., Lee C.M., Han G., Seong B.L. (2018). Glycosylation of Hemagglutinin and Neuraminidase of Influenza A Virus as Signature for Ecological Spillover and Adaptation among Influenza Reservoirs. Viruses.

[34] Chen W., Zhong Y., Qin Y., Sun S., Li Z. (2012). The Evolutionary Pattern of Glycosylation Sites in Influenza Virus (H5N1) Hemagglutinin and Neuraminidase. PLoS ONE.

[35] Na-Ek P., Thewsoongnoen J., Thanunchai M., Wiboon-Ut S., Sa-Ard-Iam N., Mahanonda R., Thitithanyanont A. (2017). The activation of B cells enhances DC-SIGN expression and promotes susceptibility of B cells to HPAI H5N1 infection. Biochem. Biophys. Res. Commun..

[36] Yu L., Shang S., Tao R., Wang C., Zhang L., Peng H., Chen Y. (2017). High doses of recombinant mannan-binding lectin inhibit the binding of influenza A(H1N1)pdm09 virus with cells expressing DC-SIGN. APMIS.

[37] Van Breedam W., Pöhlmann S., Favoreel H.W., De Groot R.J., Nauwynck H.J. (2014). Bitter-sweet symphony: Glycan–lectin interactions in virus biology. FEMS Microbiol. Rev..

[38] Thompson A.J., Cao L., Ma Y., Wang X., Diedrich J.K., Kikuchi C., Willis S., Worth C., McBride R., Yates J.R. (2020). Human Influenza Virus Hemagglutinins Contain Conserved Oligomannose N-Linked Glycans Allowing Potent Neutralization by Lectins. Cell. Host Microbe.

[39] Feinberg H., Castelli R., Drickamer K., Seeberger P.H., Weis W.I. (2006). Multiple Modes of Binding Enhance the Affinity of DC-SIGN for High Mannose N-Linked Glycans Found on Viral Glycoproteins. J. Biol. Chem..

[40] Wanzeck K., Boyd K.L., McCullers J.A. (2011). Glycan Shielding of the Influenza Virus Hemagglutinin Contributes to Immunopathology in Mice. Am. J. Respir. Crit. Care Med..

[41] York I.A., Stevens J., Alymova I.V. (2019). Influenza virus N-linked glycosylation and innate immunity. Biosci. Rep..

[42] Su S.V., Hong P., Baik S.S.W., Negrete O.A., Gurney K.B., Lee B. (2004). DC-SIGN Binds to HIV-1 Glycoprotein 120 in a Distinct but Overlapping Fashion Compared with ICAM-2 and ICAM-3. J. Biol. Chem..

[43] Marzi A., Möller P., Hanna S.L., Harrer T., Eisemann J., Steinkasserer A., Becker S., Baribaud F., Poehlmann S. (2007). Analysis of the Interaction of Ebola Virus Glycoprotein with DC-SIGN (Dendritic Cell–Specific Intercellular Adhesion Molecule 3–Grabbing Nonintegrin) and Its Homologue DC-SIGNR. J. Infect. Dis..

[44] Hong P.W.-P., Nguyen S., Young S., Su S.V., Lee B. (2007). Identification of the Optimal DC-SIGN Binding Site on Human Immunodeficiency Virus Type 1 gp120. J. Virol..

[45] Feinberg H., Mitchell D.A., Drickamer K., Weis W.I. (2001). Structural Basis for Selective Recognition of Oligosaccharides by DC-SIGN and DC-SIGNR. Science.

[46] Mitchell D.A., Fadden A.J., Drickamer K. (2001). A Novel Mechanism of Carbohydrate Recognition by the C-type Lectins DC-SIGN and DC-SIGNR. J. Biol. Chem..

[47] Appelmelk B.J., van Die I., van Vliet S.J., Vandenbroucke-Grauls C.M.J.E., Geijtenbeek T.B.H., van Kooyk Y. (2003). Cutting edge: Carbohydrate profiling identifies new pathogens that interact with dendritic cell-specific ICAM-3-grabbing nonintegrin on dendritic cells. J Immunol..

[48] Menon S., Rosenberg K., Graham S.A., Ward E.M., Taylor M.E., Drickamer K., Leckband D.E. (2009). Binding-site geometry and flexibility in DC-SIGN demonstrated with surface force measurements. Proc. Natl. Acad. Sci. USA.

[49] Guo Y., Feinberg H., Conroy E.A., Mitchell D., Alvarez R., Blixt O.E., Taylor M.I., Weis W., Drickamer K. (2004). Structural basis for distinct ligand-binding and targeting properties of the receptors DC-SIGN and DC-SIGNR. Nat. Struct. Mol. Biol..

[50] Medina R.A., Stertz S., Manicassamy B., Zimmermann P., Sun X., Albrecht R.A., Uusi-Kerttula H., Zagordi O., Belshe R.B., Frey S.E. (2013). Glycosylations in the Globular Head of the Hemagglutinin Protein Modulate the Virulence and Antigenic Properties of the H1N1 Influenza Viruses. Sci. Transl. Med..

[51] Pokidysheva E., Zhang Y., Battisti A.J., Bator-Kelly C.M., Chipman P.R., Xiao C., Gregorio G.G., Hendrickson W.A., Kuhn R.J., Rossmann M.G. (2006). Cryo-EM Reconstruction of Dengue Virus in Complex with the Carbohydrate Recognition Domain of DC-SIGN. Cell.

[52] Baribaud F., Pöhlmann S., Sparwasser T., Kimata M.T.Y., Choi Y.-K., Haggarty B.S., Ahmad N., Macfarlan T., Edwards T.G., Leslie G.J. (2001). Functional and Antigenic Characterization of Human, Rhesus Macaque, Pigtailed Macaque, and Murine DC-SIGN. J. Virol..

[53] Hoffmann E., Neumann G., Kawaoka Y., Hobom G., Webster R.G. (2000). A DNA transfection system for generation of influenza A virus from eight plasmids. Proc. Natl. Acad. Sci. USA.

[54] Uchida Y., Takemae N., Saito T. (2014). Application of Reverse Genetics for Producing Attenuated Vaccine Strains against Highly Pathogenic Avian Influenza Viruses. J. Veter. Med. Sci..

[55] Liao C.-F., Wang S.-F., Lin Y.-T., Ho D.D., Chen Y.-M.A. (2011). Identification of the DC-SIGN-Interactive Domains on the Envelope Glycoprotein of HIV-1 CRF07_BC. AIDS Res. Hum. Retrovir..

[56] Wang S.-F., Tsao C.-H., Lin Y.-T., Hsu D.K., Chiang M.-L., Lo C.-H., Chien F.-C., Chen P., Chen Y.-M.A., Chen H.-Y. (2014). Galectin-3 promotes HIV-1 budding via association with Alix and Gag p6. Glycobiology.

[57] Chang Y.-F., Wang W.-H., Hong Y.-W., Yuan R.-Y., Chen K.-H., Huang Y.-W., Lu P.-L., Chen Y.-H., Chen Y.-M.A., Su L.-C. (2018). Simple Strategy for Rapid and Sensitive Detection of Avian Influenza A H7N9 Virus Based on Intensity-Modulated SPR Biosensor and New Generated Antibody. Anal. Chem..

[58] Portolano N., Watson P.J., Fairall L., Millard C.J., Milano C.P., Song Y., Cowley S.M., Schwabe J.W.R. (2014). Recombinant Protein Expression for Structural Biology in HEK 293F Suspension Cells: A Novel and Accessible Approach. J. Vis. Exp..

[59] Payungporn S., Chutinimitkul S., Chaisingh A., Damrongwantanapokin S., Buranathai C., Amonsin A., Theamboonlers A., Poovorawan Y. (2006). Single step multiplex real-time RT-PCR for H5N1 influenza A virus detection. J. Virol. Methods.

[60] Crowley L.C., Marfell B.J., Scott A.P., Waterhouse N. (2016). Quantitation of Apoptosis and Necrosis by Annexin V Binding, Propidium Iodide Uptake, and Flow Cytometry. Cold Spring Harb. Protoc..

[61] WHO (2002). WHO Manual on Animal Influenza Diagnosis and Surveillance.

