# Supplementary material for: Identification of Important N-Linked Glycosylation Sites in the Hemagglutinin Protein and Their Functional Impact on DC-SIGN Mediated Avian Influenza H5N1 Infection
Source: Int J Mol Sci. 2021 Jan 13;22(2):743. doi: 10.3390/ijms22020743 (PMC7828482; doi:10.3390/ijms22020743)
Supplement: Supplementary file 1 [file ijms-22-00743-s001.pdf]

Supplementary Materials

Results:

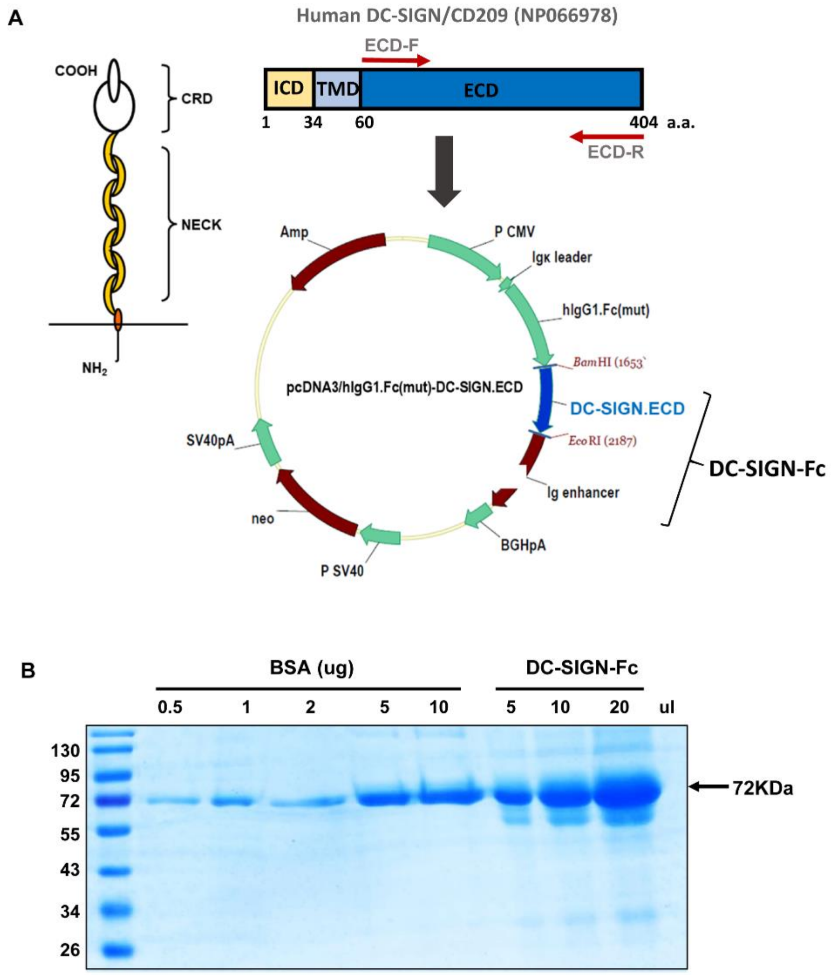

Figure S1. Generation and purification of recombinant DC-SIGN-Fc proteins. (A) The structure of DC-SIGN and map of the cloning of DC-SIGN extracellular interacting domain (ECD) to pcDNA3/hIgG-Fc(mut) plasmid (upper part). The primers used for amplification of DC-SIGN.ECD including ECD-F′: 5′-GGATCCGTCCAAGGTCC CCAGCTCCA-3′ and ECD-R′:5′-GAATTCCTACGCAGGAGGGGGGTTT-3′. (B) The purified DC-SIGN ECD protein from 293F cells were subjected to SDS-PAGE and Coomassie blue staining (lower part). The predicted size of recombinant DC-SIGN-Fc protein is 67KDa, however, the protein size detected in SDS-PAGE is around 72KDa.

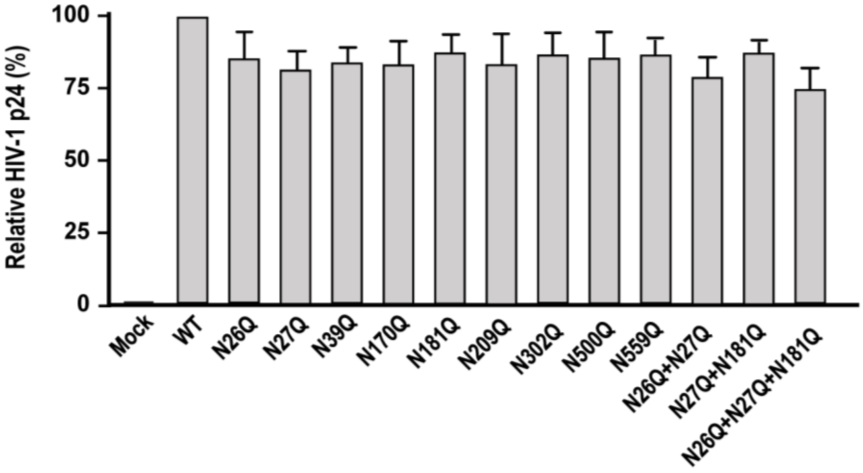

Figure S2. Determination of binding abilities of H5N1-PVs carrying N-glycosylation mutations to sialic acid expressing A549 cells. Viral binding assay was conducted using A549 cells coated ELISA and then incubated with the H5N1-PVs carrying different N-glycosylation mutants. The relative p24 values were calculated via normalizing with wild-type control.

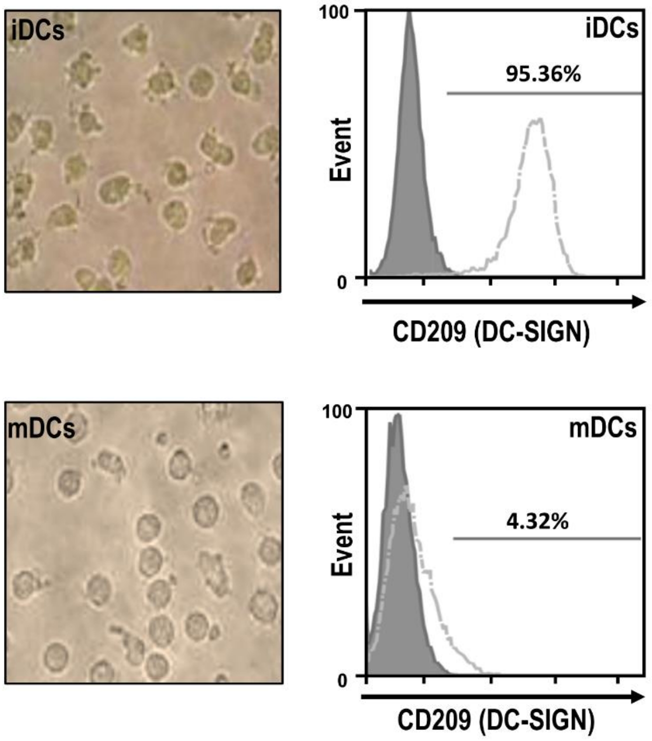

Figure S3. Generation of monocyte derived mature and immature DCs. Monocyte derived mature and immature DCs were generated and observed the morphologies under a light microscope. iDCs and mDCs were stained with anti-DC-SIGN monoclonal antibodies and subjected to FACS.
